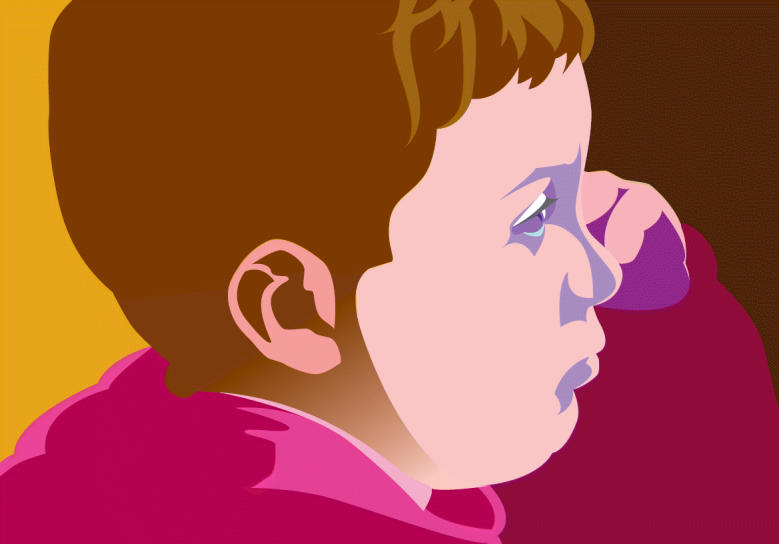# Headliners: Autism: Misfolded Protein Presents Potential Molecular Explanation for Autism Spectrum Disorders

**Published:** 2006-07

**Authors:** Jerry Phelps

De Jaco A, Comoletti D, Kovarik Z, Gaietta G, Radiæ Z, Lockridge O, et al. 2006. A mutation linked with autism reveals a common mechanism of endoplasmic reticulum retention for the α,β-hydrolase fold protein family. J Biol Chem 281:9667–9676.

Currently, there is only very limited information available on the etiology and biological basis of the autism spectrum disorders, although a mutation in the *neuroligin 3* gene has caught researchers’ attention in recent studies. Now NIEHS grantees Mark H. Ellisman and Palmer Taylor at the University of California, San Diego, and their colleagues have determined that homologous mutations in the genes coding the proteins butyrylcholinesterase (BChE) and acetylcholinesterase (AChE) cause defects in protein expression similar to those seen with *neuroligin 3*, shedding further light on a potential molecular mechanism underlying autism.

The neuroligins, BChE, and AChE are members of the α,β-hydrolase fold family of proteins. The *neuroligin 3* mutation, an arginine-to-cysteine substitution, was identified in a set of twins and has been shown to result in most of the expressed protein being retained within the endoplasmic reticulum. The small amount of protein that does reach the surface of the cell shows little binding affinity for its partner, β-neurexin, suggesting possible misfolding of the protein. Misfolded proteins are known to cause endoplasmic reticulum stress. This, in turn, can trigger cell death and contribute to human diseases including neurodegeneration, heart disease, and diabetes mellitus.

In the current study, the researchers used confocal fluorescence microscopy and analysis of oligosaccharide processing to observe whether an arginine-to-cysteine mutation affected AChE and BChE similarly despite the proteins having differing oligomerizing capacities. By inserting homologous mutations in the AChE and BChE cDNAs, they found that the mutation also resulted in endoplasmic reticulum retention of the two cholinesterases. The proteins were then likely degraded in the proteasome. The authors speculate that altering intracellular oxidation/reduction parameters may assist in the proper folding and export of these proteins.

## Figures and Tables

**Figure:**